# Supplementary material for: Adopting Andersen’s behavior model to identify factors influencing maternal healthcare service utilization in Bangladesh
Source: PLoS One. 2021 Nov 29;16(11):e0260502. doi: 10.1371/journal.pone.0260502 (PMC8629289; doi:10.1371/journal.pone.0260502)
Supplement: Attachment — Submitted filename: Adopting MHC.docx [file pone.0260502.s002.docx]

**Adopting Andersen’s behavior model of health service use to identify factors influencing maternal healthcare service utilization in Bangladesh**

This is a good research work. However I have mentioned below very few recommendation

**Title-** Extra word used in title. It needs to be more scientific/ research title. Type of research method need to be mentioned

**Abstract-** Objective is not addressed properly as mentioned in the title. The method is not completely mentioned. The process of data collection and analysis is not mentioned. The grammar/ tense need to be correct in result section. What is Andersen's model need to be mentioned? Factors are not addressed properly in result section.

**Introduction-** Andersen's model related information need to be added. Starting sentence/ para is not attractive. The problem is not addressed adequately.

**Method-** Method section need to be concise. The ethical approval, conflict of interest, limitation of the study is not mentioned. The data extraction process need to be clearly mentioned.

**Result-** Section is OK, but as mentioned table in BDHS report is similar in this manuscript, then need to be changed.

**Discussion-** Discussion section is large enough, need to be concise. First para should contain the major findings of this research. How other research similar or dissimilar with this findings need to be mentioned properly.
