# Supplementary material for: Adopting Andersen’s behavior model to identify factors influencing maternal healthcare service utilization in Bangladesh
Source: PLoS One. 2021 Nov 29;16(11):e0260502. doi: 10.1371/journal.pone.0260502 (PMC8629289; doi:10.1371/journal.pone.0260502)
Supplement: Attachment — Submitted filename: Review of Manuscript - ABM - Recommendations.docx [file pone.0260502.s003.docx]

Recommendation (Accept, Minor Revision, Major Revision, Reject) : Minor revision
1. Is the manuscript technically sound, and do the data support the conclusions? (Answer options: Yes, No, Partly) : Yes
2. Has the statistical analysis been performed appropriately and rigorously? (Answer options: Yes, No, I don’t know, N/A): Yes
3. Does the manuscript adhere to the PLOS Data Policy? Additional details can be found at <http://journals.plos.org/plosone/s/materials-and-software-sharing>. : Yes
4. Is the manuscript presented in an intelligible fashion and written in standard English? (Answer options: Yes, No): Yes
5. Review Comments to the Author:

The manuscript is well written, technically sound and backed by analysis of data used from the secondary source BDHS which is available. The author has concluded mentioning the broader issues to address such as poverty, geographical disparity and in health services. However, if there was a scope to further examine the influence of having maternal health scheme or health protection schemes which are currently being tested in few districts of Bangladesh and that could give a strategic direction to the policy makers to find the desirable use of MHS in Bangladesh.

The methodology was well articulated and the adaptation of HBM with categorization of accessibility issues of MHS services has been illustrated adequately. The sampling was done following standard process and statistical analysis was done with rigor. The tables and analysis have been helpful to understand the logic and inference drawn by author. Regarding data availability and all the analysis, the authors has ensured that all data underlying the findings described in their manuscript are fully available without restriction. To my knowledge and understanding the author has written the manuscript in an intelligible fashion and the article is clear and correct without any ambiguity.

As an additional comments to author, author could review literature available specifically icddr,b which has many publications in relation the MNCH services ( reference) as they have been doing longitudinal study using their Matlab database. The other important issue that author could consider during sampling, one or two district's BDHS data could be purposively considered for analysis by using the adapted HBM framework to have a comparative picture of factors that influences which the author mentioned in his conclusion. The purposive sampling of these special districts is currently having government's (MOHFW and Health Economic Department) maternal health scheme and health protection scheme, and this is done to test to address equity issues in health especially in maternal health. The government's schemes will be adapted to achieve UHC in near future. Therefore, this was missed opportunity that could have been considered or may be in future studies to help policy makers and planners. Apart from this I do not have any concerns or specific comments.

6. If you would like your identity to be revealed to the Authors, please list your name.
7. Dr. Munir Ahmed

Confidential to Editor:
1. Do you have any potentially competing interests? If none, type "None." Our policy on competing interests can be found at <http://journals.plos.org/plosone/s/competing-interests>.: None
2. Did you receive any assistance in preparing this review (e.g. from a post-doc or graduate student)? If yes, please provide his/her name and title. NO

If you have chosen to reveal your identity to the authors in question 7 above, please include the name of the person who assisted you in the response to question 7 as well. (optional) : NA
3. Yes, and Homepage

Orchid review credit: Yes
